# Planktonic microbial profiling in water samples from a Brazilian Amazonian reservoir

**DOI:** 10.1002/mbo3.523

**Published:** 2018-01-30

**Authors:** Bianca C. A. Cabral, Luísa Hoffmann, Bruce Budowle, Turán P. Ürményi, Rodrigo S. Moura‐Neto, Sandra M. F. O. Azevedo, Rosane Silva

**Affiliations:** ^1^ Instituto de Biofísica Carlos Chagas Filho Universidade Federal do Rio de Janeiro Rio de Janeiro Brazil; ^2^ Center for Human Identification University of North Texas Health Science Center TX USA; ^3^ Center of Excellence in Genomic Medicine Research (CEGMR) King Abdulaziz University Jeddah Saudi Arabia; ^4^ Instituto de Biologia Universidade Federal do Rio de Janeiro Rio de Janeiro Brazil

**Keywords:** archaea, betaproteobacteria, cyanobacteria, massively parallel sequencing, microbial mapping, phage

## Abstract

Our comprehension of the dynamics and diversity of freshwater planktonic bacterial communities is far from complete concerning the Brazilian Amazonian region. Therefore, reference studies are urgently needed. We mapped bacterial communities present in the planktonic communities of a freshwater artificial reservoir located in the western Amazonian basin. Two samples were obtained from rainy and dry seasons, the periods during which water quality and plankton diversity undergo the most significant changes. Hypervariable 16S rRNA and shotgun sequencing were performed to describe the first reference of a microbial community in an Amazonian lentic system. Microbial composition consisted mainly of Betaproteobacteria, Cyanobacteria, Alphaproteobacteria, and Actinobacteria in the dry period. The bacteria distribution in the rainy period was notably absent of Cyanobacteria. Microcystis was observed in the dry period in which the gene cluster for cyanotoxins was found. Iron acquisition gene group was higher in the sample from the rainy season. This work mapped the first inventory of the planktonic microbial community of a large water reservoir in the Amazon, providing a reference for future functional studies and determining other communities and how they interact.

## 1 INTRODUCTION

The understanding of the composition and dynamics of water microbial community is essential to promote knowledge on ecological processes of the ecosystem. Microbiota constitutes one of the most important compartments of carbon metabolism in aquatic environments and also plays a central role in the nutrient recycling process. However, knowledge about the importance of these aquatic microbial communities on the dynamics of aquatic communities and the diversity of these microbial assemblies in time and space is scant especially regarding the lack of studies on tropical freshwater ecosystems. The increasing number of artificial reservoirs may affect this ecosystem. The always‐wet rainforest is considered the richest ecosystem in species on earth, but very few studies have attempted to describe microbial communities in the Amazon basin. Culture‐independent metagenomic processing of samples is an excellent tool for community characterizations, which range from the analysis of whole genomes to selected genes. Analysis of this type includes one random sequencing study in the Amazon river (Ghai et al., 2011) and one of the Tucurui hydropower plant using cloning methods (Graças et al., 2011). Moreover, the understanding of phytoplankton dynamics and those of aquatic bacteria in Amazonian lentic systems is poorly evaluated especially the relationship with the heterotrophic microbial community. The Samuel hydroelectric plant reservoir, located in Rondonia State in the western Amazonian basin, was built and flooded an area covered by a secondary forest, pastures, and small cultivated land. The flood area is 584.26 km^2^, with a flow rate of 265 m^3^ s^−1^, water residence time of 105 days and annual mean water temperature about 30°C. The reservoir microbial community in this hot and rainfall‐dependent system has yet to be described. The analysis reported herein is 16S rRNA, and shotgun analysis by massively parallel sequencing (MPS) High‐throughput sequencing methods were selected to reduce bias interpretation and lack of information on the overall community due to previous cloning‐based studies. These results may contribute to understanding bacterial community diversity during the main Amazonian seasons: dry and rainy periods.

## 2 MATERIALS AND METHODS

### 2.1 Sample collection

**Figure 1:**
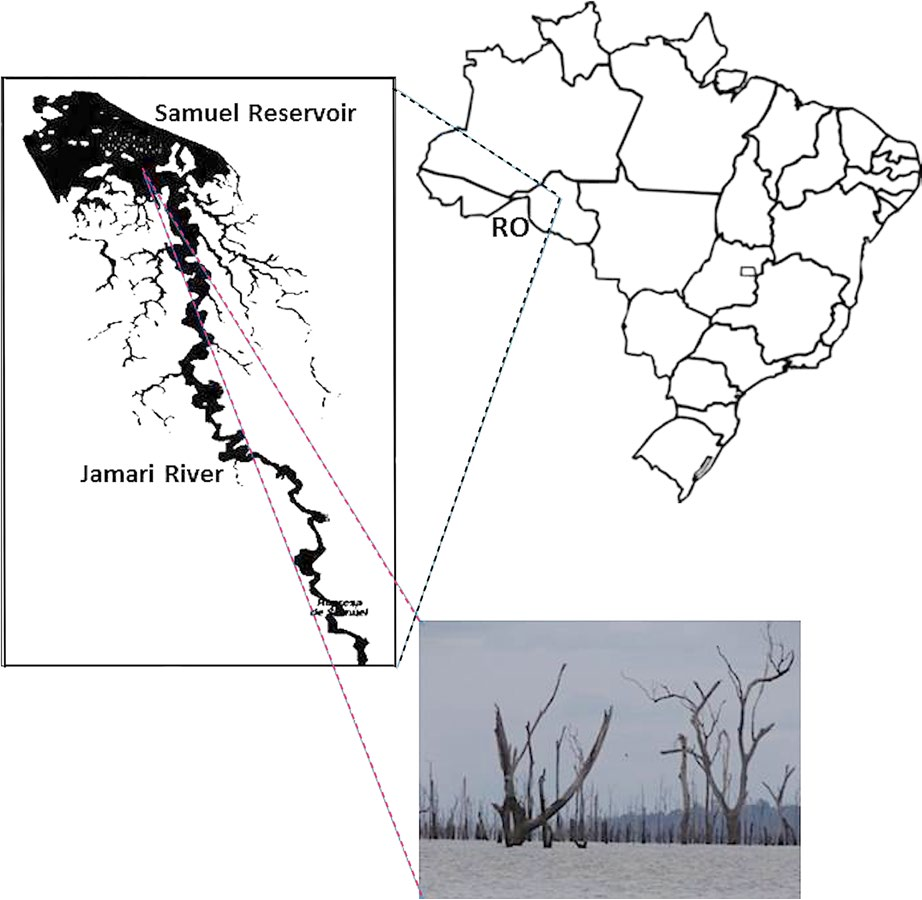
Spatial illustration of Samuel hydroelectric power plant located in the state of Rondonia (08° 45′ 02.6″ S and 63° 26′ 25.9″ W), Amazon region, Brazil. The picture was taken on 23 October 2013 illustrates the tree remains in the water reservoir

The Samuel reservoir belongs to the hydroelectric power plant located in the State of Rondônia (08° 45′ 02.6″ S and 63° 26′ 25.9″ W). It is an oligotrophic reservoir, which was formed by the Jamari River in Northwestern Brazil (Figure 1). The total area is approximately 634 km^2^, the depth reaches 87 m, the average width is 20 km, and the maximum length is 210 km (Fearnside, 2005; Lima, 2005). In the Amazon rain forest, the dry and rainy periods are indicated by the amount of rainfall. In the Amazonian reservoir, the main driving force that induces seasonal variation on the phytoplankton community is the annual cycles of a wet and dry period. There are periods of nutrients enrichment due to external loading and more turbulence during the rainy season. Sample collection was performed on 26 June 2013 and 23 October 2013 from the water surface. The June sample represents the dry season period, and the October sample represents the rainy season period (Alvares, Stape, & Sentelhas, 2013). Water samples were collected near the dam using a 20 μm mesh phytoplankton net with 30 cm of diameter and 70 cm in length, hooked horizontally to a slow moving (≅3.6 km/hr) motorboat in a circle, for 5 min. Water temperature, conductivity, oxygen content, and pH were measured using a YSI 600QS multiparametric probe (YSI Inc., Yellow Springs, OH, US). The sample, volume of approximately 150 ml, collected in a PVC cylinder, was refrigerated and subsequently frozen and maintained at −20°C. Alternatively, another water collection method was performed for the 23 October 2013 sampling. We pooled the collected material through the 20 μm mesh phytoplankton net for every 50 cm from the euphotic zone (determined as 2.7‐times the Secchi disk depth). No specific permits were required, and no protected or endangered species were disturbed during the sampling activities.

### 2.2 DNA extraction

The 150 ml water samples were thawed, lyophilized and then maintained at room temperature until processed. DNA was extracted from 0.25 g of the lyophilized material using the Power Soil DNA Isolation Kit (MoBio Laboratories, Carlsbad, US). DNA integrity was evaluated by agarose gel electrophoresis stained with ethidium bromide and quantity of recovered DNA was determined using a fluorometer (Qubit ‐Thermo Scientific, US) according to manufacturer's recommendations. DNA extraction from samples and library preparations were performed on separate days.

### 2.3 16S rRNA library preparation

The amplification of the 16S rRNA hypervariable regions were performed using the following primers: for V3 region: 341F 5′‐CCTACGGGAGGCAGCAG‐3′ and 518R 5′‐MTTACCGCGGCTGCTGG‐3′; V6 region: 907F 5′‐AAACTCAAATGAATTGACGG‐3′ and 1100R 5′‐AGGGTTGCGCTCGTTG‐3′ and V8 region: 1237F 5′‐GGGCTWCACACGTVMTAC‐3′ and 1391R 5′‐GACGGGCGGTGTGTAMA‐3′. F corresponds to forward and R to reverse primers. Amplification of each of the targets was performed in independent polymerase chain reactions (PCR). The PCR final volume was 50 μl and contained 0.2 mmol of each primer (Thermo Scientific), 0.1 mmol/L dNTPs (dATP, dTTP, dCTP, dGTP) (Thermo Scientific), 1.5 mmol/L MgCl_2_ (Thermo Scientific), 2.5 units (U) of Taq DNA Polymerase with the respective buffer (Invitrogen) and 24 ng of DNA. The PCR was performed in the Veriti thermocycler (Life Technologies). The amplification was conducted under the following conditions: initial denaturation at 94°C for 5 min, 30 cycles of denaturation at 94°C for 30 s, annealing at 55°C for 30 s and extension at 72°C for 30 s and a final extension at 72°C for 10 min. The amplified product was stored at −20°C. Samples containing the V3, V6, and V8 amplified fragments were purified using magnetic beads Agencourt AMPure (Beckman Coulter, USA) and a magnetic stand (Life Technologies, US). The amplified products were quantified using a Qubit^®^ fluorometer (Invitrogen, US). Libraries were constructed from 100 ng of the amplified product using Ion Fragment Library Kit Plus (Life Technologies), following the manufacturer's protocols.

### 2.4 Shotgun library preparation

Metagenomic DNA was enzymatically fragmented using Ion Shear DNA kit, and the sequencing library was prepared using Ion Xpress™ Plus Fragment Library Kit (Thermo Fisher Scientific, US) with barcodes (Ion Xpress™ Barcode Adapters kit, Thermo Fisher Scientific, US). The Libraries were quantified using the Ion Library TaqMan^®^ Quantitation kit.

### 2.5 Massively parallel sequencing

Libraries were appropriately diluted for clonal amplification of Ion Sphere™ Particles (ISPs) by emulsion PCR using the Ion PGM Template OT2 400 kit and subsequently enriched. Sequencing was performed on a 318 chip in an Ion Torrent Personal Genome Machine (Thermo Fisher Scientific, USA). Length and quality filters were applied on the reads by the Torrent Suite v4.0 software (Thermo Fisher Scientific, US).

### 2.6 Sequence read processing and annotation of metagenomic and 16SrRNA profiles

Sequence data were exported in FASTQ format by the Torrent Suite software and uploaded to MG‐RAST (Metagenomics Analysis) server on November 2014. Reads from the three regions of the16SrRNA from both samples were processed by the NGS analysis pipeline of the SILVA rRNA gene database project (SILVAngs 1.3) (Quast et al., 2013), according previous work (Ionescu et al., 2012). Processing of shotgun reads was performed using the metagenomics pipeline (Meyer et al., 2008) with default options for initial quality control (QC) filtering of raw reads. Low‐quality and duplicated sequences were removed. The remaining sequences were matched to the M5NR (Wilke et al., 2012) database for organism classification and functional categories identification using SEED subsystem. This system is based on genomic annotation data that are curated in a set of functional roles (Overbeek et al., 2014). The maximum *e*‐value thresholds of 1.0e–05, minimum alignment length of 50 bp and minimum percentage of identity cutoff of 60% were used for functional annotation and classification. Identification of gene clusters from secondary metabolites was accomplished using Anti‐Smash (Antibiotics and Secondary Metabolite Analysis Shell) (Medema et al., 2011). CLC Genomic Workbench software (v.8.5.1) was used for sequencing comparison and alignments. Assembly reads to a contig were performed using the parameters: Gap open cost = 10, gap extension = 1. NJ tree was constructed using Jukes Cantor as evolution model and 100 boostrap analysis.

## 3 RESULTS

### 3.1 Sample collection and water profiling

Samples were collected from the Samuel reservoir of the northwestern Amazonian basin during the rainy and dry seasons to perform an initial assessment of the aquatic microbial communities with season variation in the aquatic microbial profile. Small differences in biochemical characteristics among the water samples were observed (Table 1). The rainy period sample (200 mm average rainfall/month) showed slightly elevated O_2_, less water conductivity and pH around seven compared with the characteristics of the dry period sample (70 mm average rainfall/month).

**Table 1:** Physical and chemical water parameters

Samuel hydroelectric power plant reservoir		
	Collection date	Collection date
	26 June, 2013 (dry‐period sample)	23 October, 2013 (rainy‐period sample)
pH	7.6	6.9
Conductivity (μS/cm)	23	19
O_2_ (mg/L)	7.8	9.2
Temperature (°C)	30.0	30.7
Rainfall (mm)/month	70	200

### 3.2 Taxonomical annotation and classification

**Figure 2:**
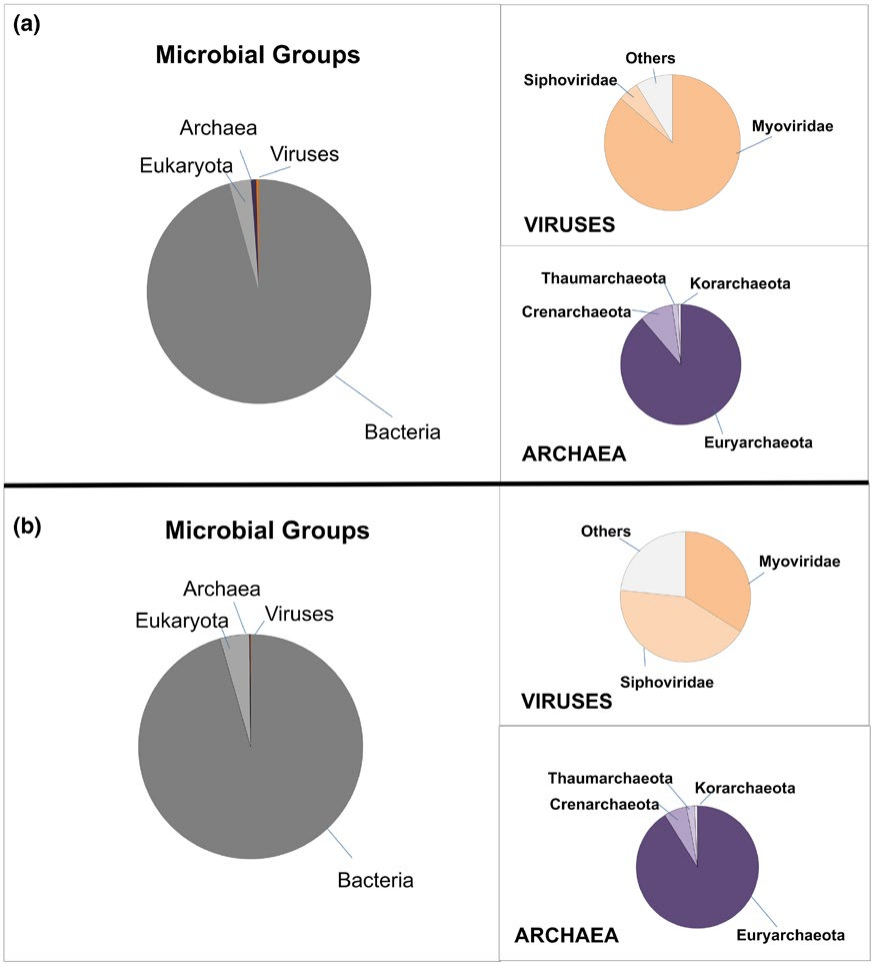
Relative abundances of Bacteria, Eukarya, Family of viruses and Archaea genera distribution according to season period. (a) Dry period, (b) Rainy period

Reads from shotgun library were grouped according to taxonomic classification to obtain the domain distributions within the samples collected from the Samuel Reservoir. The results found in the dry and rainy period samples showed that bacteria was the most common cellular domain lineage: 96% and 94% (Figure 2a and b), followed by Eukarya (2.6% and 5.4%), Archaea (0.7 and 0.1%); and viruses (0.4% and 0.1%), respectively. The majority of the reads associated with Eukarya were unassigned. The genera of Archaea found in the dry and rainy period samples were the methane producers: 86% and 67% and mainly Methanosarcina 36% and 39%, respectively (Figure 2a and b). The distribution of virus families in the dry period sample was Myoviridae (87%) followed by Siphoviridae (5%) (Figure 2a). This proportion changed in the rainy period sample, as follows: Siphoviridae 43% and Myoviridae 34%, and unknown viruses were around 23% (Figure 2b).

**Figure 3:**
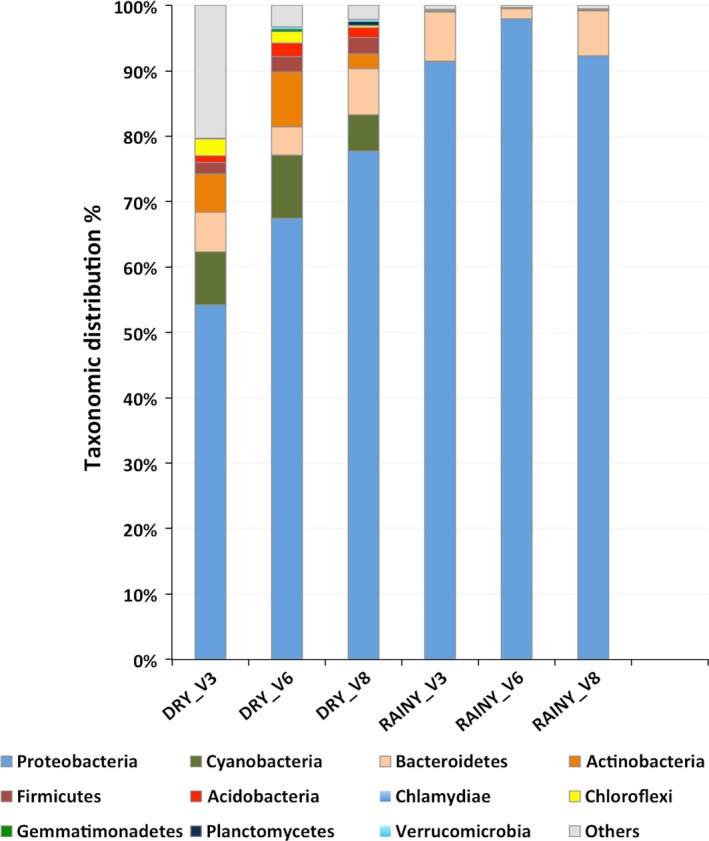
Relative abundances of the bacterial phyla profile from the dry and rainy period sample of Samuel reservoir based on the 16SrRNA fragments analyzed by SILVAngs. Others represent minor relative proportions of bacterial phyla and no relative reds

Sequencing from the three hypervariable regions of 16S rRNA (V3, V6, and V8) were obtained from both samples. Reads from 16SrDNA fragments were grouped according SILVAngs classification (Data S1). Bacterial phyla detected in both samples are very distinct regardless the 16SrRNA fragment (Figure 3). Proteobacteria was the major component in the dry (V3: 54%; V6: 67%; and V8: 77%) and rainy samples (V3: 91%; V6: 97% and V8: 92%). Also Cyanobacteria (V3: 8%; V6: 9%; V8: 6%), Bacteroidetes (V3: 6%; V6: 4%; V8: 7%), and Actinobacteria (V3: 6%; V6: 8%; V8: 2%) were present in the dry sample. In contrast, Bacteroidetes (V3: 7%; V6: 2%; V8: 7%) were the second major phylum in the rainy period sample.

**Figure 4:**
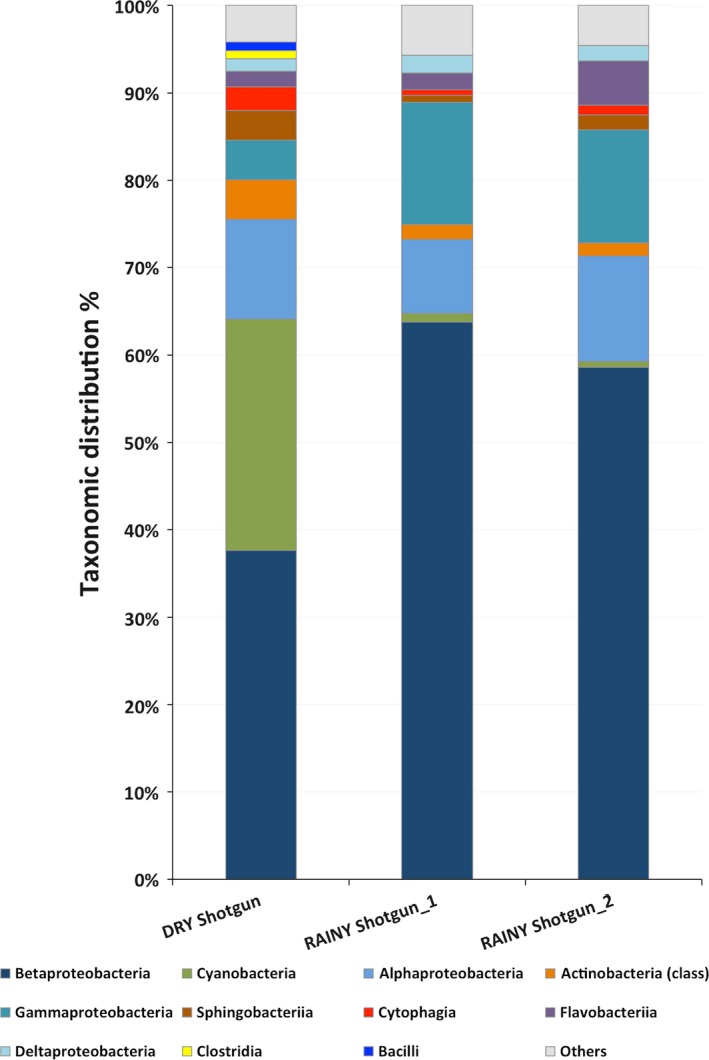
Relative abundances of the bacterial profile according dry and rainy period samples of Samuel reservoir based on the shotgun sequence analysis using MG‐RAST. DRY shotgun and RAINY shotgun_1 samples were collected from the water surface. RAINY shotgun_2 water sample was collected every 50 cm from the euphotic zone. Others represent minor relative proportions of bacterial class. Unassigned reads were not considered in the comparison

Metagenomic analysis using random sequencing ‐shotgun **(**Data S2) was performed to provide a confidence map of the taxonomic groups of Bacteria. Figure 4 shows the bacterial classes that were detected in samples by shotgun (DRY shotgun and RAINY shogun 1). We also compared microbial distribution in a rainy period by integrating water collected from every 50 cm from the euphotic zone (Figure 4_RAINY shotgun 2). The bacterial profile distribution among samples revealed that Betaproteobacteria was the most prevalent class in both samples representing 37% in the dry period and 64% (shotgun 1) and 58% (shotgun 2) in the rainy period sample. The second most abundant taxon was Cyanobacteria in the dry period sample (26%), but was negligible in the rainy period sample (<1%). In the dry period sample, the minor contributors were Alphaproteobacteria (11%), Actinobacteria (5%), Gammaproteobacteria (5%), Sphingobacteria (3%), Cytophagia (3%), Flavobacteriia (2%), Bacilli (1%), Deltaproteobacteria (1%), and Clostridia (1%). Nevertheless, in the rainy period samples in addition to the predominant Betaproteobacteria, we found Gammaproteobacteria (shotgun 1: 14%; shotgun 2: 13%), Alphaproteobacteria (shotgun 1: 9%; shotgun 2: 12%), Deltaproteobacteria (shotgun 1 and 2: 2%), Flavobacteriia (shotgun 1: 2%; shotgun 2: 5%), Actinobacteria (shotgun 1 and 2: 2%), Sphingobacteriia (shotgun1 and 2 1%), Cytophagia (shotgun 1: 0.7%; shotgun 2: 1%), and Cyanobacteria (shotgun 1 and 2: <1%). These results confirmed the relative abundance of the microbial community and the low abundance of Cyanobacteria in the two independent sample collections. Cyanobacteria richness can affect human health, and some genera can be toxin producers. A higher prevalence (6.5%) of the genus from Cyanobacteria Microcystis, Cyanothece sp. (1.3%), Nostoc (1.1%), Synechocystis (0.9%), and Anabaena (0.8%) were observed in the dry period sample (Table 2). Betaproteobacteria genera (Methylibium, Leptothrix, Polaromonas, Acidovorax, Janthinobacterium, Herbaspirillum, Albidiferax, Variovorax, Herminiimonas, Ralstonia, Chromobacterium, Dechloromonas) were also present. Likewise, these genera were found in the rainy period sample, but Janthinobacterium was more abundant (9.8%) followed by Herbaspirillum (7.7%) and Herminiimonas (6.8%). These relative abundances of bacterial composition suggest potential differences in functional gene composition in the two samples.

**Table 2:** Relative abundances of the bacterial genera distribution among the dry or rainy period samples based on the shotgun sequence reads using MG‐RAST

DRY period sample			RAINY period sample		
Phylum/class	Genus	%	Phylum/class	Genus	%
Betaproteobacteria	***Methylibium***	2.0	Betaproteobacteria	***Janthinobacterium***	**9.8**
	***Leptothrix***	1.7		***Herbaspirillum***	**7.7**
	***Polaromonas***	1.5		***Herminiimonas***	**6.8**
	***Acidovorax***	1.4		*Burkholderia*	1.1
	***Janthinobacterium***	1.2		*Cupriavidus*	2.6
	***Herbaspirillum***	1.0		***Ralstonia***	2.1
	***Albidiferax***	0.8		***Polaromonas***	1.5
	***Variovorax***	0.8		***Acidovorax***	1.4
	***Herminiimonas***	0.8		*Oxalobacter*	1.4
	***Ralstonia***	0.7		***Albidiferax***	1.1
	***Chromobacterium***	0.6		***Variovorax***	1.0
	***Dechloromonas***	0.6		***Chromobacterium***	0.9
	*Verminephrobacter*	0.5		***Methylibium***	0.8
Cyanobacteria	*Microcystis*	**6.4**		*Nitrosospira*	0.7
	*Cyanothece*	1.3		***Dechloromonas***	0.7
	*Nostoc*	1.1		*Aromatoleum*	0.6
	*Synechocystis*	0.9		*Azoarcus*	0.6
	*Anabaena*	0.8		***Leptothrix***	0.6
Alphaproteobacteria	*Bradyrhizobium*	1.6		*Delftia*	0.5
	*Rhodopseudomonas*	1.2	Gammaproteobacteria	*Pseudomonas*	2.7
Flavobacteriia	***Flavobacterium***	0.5		*Xanthomonas*	0.6
Deltaproteobacteria	*Anaeromyxobacter*	1.0	Flavobacteriia	***Flavobacterium***	0.5
Sphingobacteriia	*Chitinophaga*	1.1			
Others (<0.5%)		70.5	Others (<0.5%)		54.3

Genera, in bold, were found in both samples; bold numbers represent the most abundant genus in dry and rainy period samples. Only Genera found >0.5% in the samples are displayed.

### 3.3 Comparison of functional gene categories

**Figure 5:**
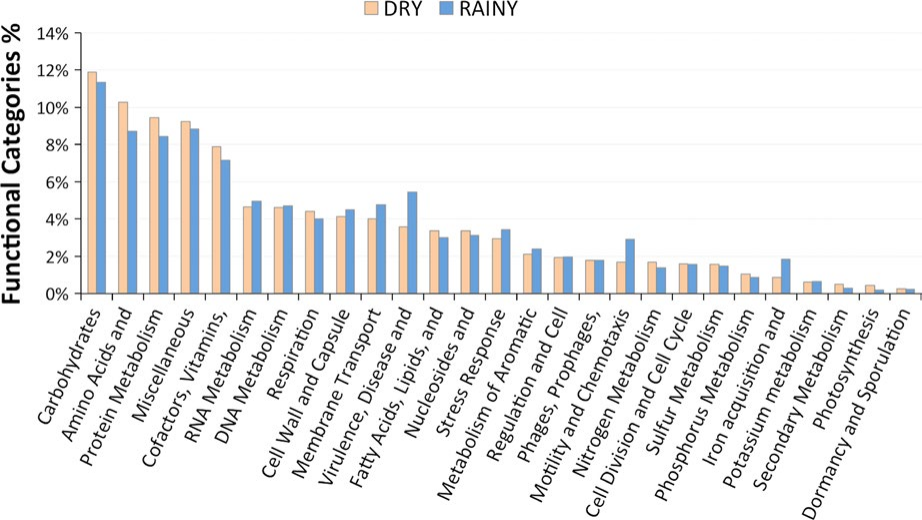
Relative abundance of gene functional categories in the rainy and dry period samples

Information on the metabolic capacity present in the samples was computed by gene predictions assigned by SEED subsystems. All functional system categories analyzed were present in both samples. Functional gene categories such as carbohydrate metabolism, amino acid and protein metabolism, respiration, sporulation and dormancy, cell division, and others were found in both samples (Figure 5). Genes involved in virulence, motility and iron metabolism were present in a higher proportion in the rainy period sample. The rainy period conditions might favor species of Betaproteobacteria that metabolize iron. In contrast, high content of Cyanobacteria that produce cyanotoxin can be an obstacle to the human use of the water resource. Since Microcystis accounted for 6% of the total genera in the dry sample, we investigated specific gene clusters involved in secondary metabolites. To this purpose, all the reads were assembled into contigs (Data S2) and searched for gene clusters of nonribosomal peptide synthetases of microcystins. We found partial coding sequences for known genes, such as the microcystins (McyC) (Data S2), nonribosomal peptide synthetase, cyanobactin (peptidase S8). Also cyanopeptin (McnC) and aeruginosin (AerG1) gene clusters and the ribosomal synthesis as bacteriocin (acetyltransferase), microviridin (methyltransferase) (data not shown). These results suggest that bacterial species, likely driven by the environmental conditions of the two periods, shifted their abundance in the reservoir.

## 4 DISCUSSION

The consequences of periods of heavier rainfall are crucial for Amazonian basin dynamics (Malhi et al., 2008). Small variations in water conditions such as conductivity, O_2_, and pH, can alter the microbiome in this reservoir. Physical and chemical parameters of the water samples were in agreement with previous studies performed in the area around the year. The water temperature of 30°C of the reservoir at the collection periods was constant throughout the year (Alvares et al., 2013). Bacteria were the main microorganisms found in both samples. The unassigned Eukaryote reads may be due to the smaller number of sequences from eukaryotic planktonic organisms in the available databases. Phages were found in both samples probably as particles attached to cells and not as free‐living particles. Bacteriophages have been reported in different environments (Srinivasiah et al., 2008) and associated with almost all recognized bacterial taxa. The majority of Cyanophages belonging to the family Myoviridae was found in the dry period sample in which Cyanobacteria were abundant. Myoviridae, a well‐known Cyanobacteria phage, was also observed in another reservoir dam in Poland (Mankiewicz‐Boczek et al., 2016), possibly indicating broad geographic distribution. (Huang, Wang, Jiao, & Chen, 2012; Mankiewicz‐Boczek et al., 2016; Roux et al., 2012). Bacteriophages may regulate the environment by the cycling of organic matter in the biosphere and influence the evolution of bacterial genomes and pathogenicity or antibiotic resistance (Balcazar, 2014; Chibani‐Chennoufi, Bruttin, & Dillmann, 2004). Another element in the environmental microbial community is Archaea that usually is present in low abundance compared with Eubacteria. In our data, there were small portions of Archaea, most of them were methanogens in dry and rainy period samples. This result may indicate that they are contributing to the degradation of organic matter in the water column, since the methane emissions from this hydroelectric reservoir releases, in average, was reported as 71.19 ± 107.4 mg CH4/m^2^ d^−1^ (Lima, 2005). Nevertheless, the Archaeal community composition did not vary in our samples. It was reported that in different Alpine stagnant water ponds, compared to flowing freshwaters during summer, showed significantly higher abundances and diversities of Archaeal communities, especially methanogens (Hedrich & Schlomann, 2011). However, Methanosarcina was not observed in a tropical estuarine bay (Vieira et al., 2007). The increased amount of Cyanobacteria in the dry period sample in the shotgun analysis was also detected using 16S rRNA. Relative differences in taxa abundance could be due to bias in amplification of Cyanobacteria with the primers designed for the three hypervariable regions of the 16S rRNA. It was reported that primers might be specific for some species or a combination of a template in the amplification reaction (Barriuso, Valverde, & Mellado, 2011; Chakravorty, Helb, Burday, Connell, & Alland, 2007). Previous studies by our group on this reservoir identified and isolated at least three different strains of Microcystis aeruginosa. All of them are microcystins producers (unpublished results). Cyanobacteria dominance (Microcystis sp., Cyanothece sp., Nostoc sp., Synechocystis sp., Anabaena sp.) in an oligo‐mesotrophic reservoir located in a region where anthropogenic impacts are increasing fast and without any actual control may trigger environmental damage and human health problems. Heterotrophic bacteria were present in different proportions in both samples. The dynamics of heterotrophic bacteria progression may affect Cyanobacteria by enhancing their growth as suggested by other groups (Berg et al., 2009; Louati et al., 2015; Xie et al., 2016). Also, cyanotoxin production usually follows the cyanobacterial cell density. Our results are in agreement with studies on blooms of the genera Anabaena and Microcystis (Berg et al., 2009) that were accompanied by the relative abundance of Betaproteobacteria and Alphaproteobacteria. This cooperative relationship includes strains that are capable of degrading cyanotoxins or organic compounds, which could be used to assess and control the harmful properties of Cyanobacteria as described by Neilan, Pearson, Muenchhoff, Moffitt, & Dittmann (2013). In contrast, the closely related genera Janthinobacterium, Herbaspirillum and Herminiimonas belonging to Family Oxalobacteraceae were found in the rainy period sample. Burkholderiaceae was also predominant in this period, suggesting that environmental conditions favored these families.

Comparison of functional gene categories in the metagenomic samples was evaluated by SEED subsystems (Aziz et al., 2008), which is an automated server that assigns functions to the genes. Our results suggested that the photosynthesis and secondary metabolism are higher in the dry period sample. These results can be attributed to the greater abundance of Cyanobacteria in this period, increasing the photosynthesis capacity of the system. The iron acquisition, motility, and chemotaxis, as well as virulence and membrane transport, were higher in the rainy period sample. This observation may be due to a heavy runoff that is usually observed in this the high rainfall season causing discharge of the soil in the aquatic ecosystem supporting species that metabolizes iron, which is abundant in the Amazonian soil (Allard, Menguy, Salomon, & Calligaro, 2004; Bergquist & Boyle, 2006). This fact may favor the increasing abundance of Betaproteobacteria in the rainy period. Some of the Betaproteobacteria have been associated with a higher capacity to absorb iron (Hedrich & Schlomann, 2011) and high metabolic profiles for carbohydrates, carboxylic acids, and amino acids (Klann, McHenry, Montelongo, & Goffredi, 2016). As already mentioned, very few studies have attempted to describe microbial communities in the Amazon basin, and especially in the growing number of artificial reservoirs that may affect this ecosystem. One study conducted in a dam located on the Tocantins River, which is approximately 2,000 km northeast of our field workplace, described a microbial abundance of Actinobacteria (56%) and Proteobacteria (30%) on the photic zone using Sanger sequencing of 16S rRNA clones (Graças et al., 2011). Another work on the Amazon River (pristine condition) sampled the water at the end of the dry season showing the abundance of Betaproteobacteria (25%), Actinobacteria (20%), Alphaproteobacteria (17%), and Gammaproteobacteria (12%) (Ghai et al., 2011). Bacterial distribution done in the Amazon Basin freshwater lakes (Lake Poraquê, Lake Preto, Manacapuru Great Lake and Lake Ananá) showed an average abundance of proteobacteria (47%), Actinobacteria (29%) Cyanobacteria (17%), Firmicutes (5%), and Bacteroidetes (3%) (Toyama et al., 2016). In the same period (September 2008) samples from Amazon Basin Riverine habitat (Solimões, Purus and Urucu Rivers) showed an average relative distribution of proteobacteria (45%), Actinobacteria (20%) Cyanobacteria (26%), Firmicutes (5%), and Bacteroidetes (5%) (Santos‐Júnior et al., 2017). These results are similar to our data in the dry period sample. Nevertheless, they investigated only the most common taxonomic groups in the Amazon Basin and there is no temporal metagenomic analysis. In summary, our work inventoried the seasonal distribution of Phages, Archaea, and Bacteria present in the Samuel Reservoir in the Amazon and discussed the gene categories function of the ecosystem.

## 5 CONCLUSION

Here we presented the reference of the microbial community from samples collected in two rainfall seasons of an artificial water lentic system in the Amazonian basin using massive parallel sequencing. This inventory may be useful to understanding the bacterial community composition during Cyanobacteria blooms and for future environmental studies. Of particular importance for the water flux in the Amazonian basin, this study added an understanding of the microbial community found in this ecosystem and might be combined to further functional studies to determine how they interact.

## DATA AVAILABILITY

The metagenomic and 16S rDNA data were stored on the MG‐RAST server under ID numbers 4601005.3; 4636739.3; 4636740.3; 4636741.3. Dataset is also available as BioProject: PRJNA390178.

## CONFLICT OF INTEREST

None declared.
