# Supplementary material for: Planktonic microbial profiling in water samples from a Brazilian Amazonian reservoir
Source: Microbiologyopen. 2018 Jan 30;7(2):e00523. doi: 10.1002/mbo3.523 (PMC5911997; doi:10.1002/mbo3.523)
Supplement: Supplementary file 1 [file MBO3-7-na-s001.docx]

**Supporting information 1**

**Raw sequencing data information**

**A) Reads from 16SrDNA fragments according SILVAngs pipeline.**

| Sample Name | Number of sequences | Avg. Length (aligned) | # OTUs |
| --- | --- | --- | --- |
| DRY_V3 | 134,281 | 115 | 17,318 |
| DRY_V6 | 166,771 | 139 | 29,303 |
| DRY_V8 | 40,544 | 154 | 13,012 |
| RAINY_V3 | 290,380 | 166 | 18,652 |
| RAINY_V6 | 70,818 | 147 | 4,277 |
| RAINY_V8 | 286,208 | 165 | 21,936 |
| Project UHE-Samuel | 989,002 | 152 | 104,498 |

**B) Metagenomic reads according MG-RAST pipeline**

| **Parameters** | **June 2013 dry** | **Oct 2013 rainy_shotgun 1** | **Oct 2013 rainy_shotgun 2** |
| --- | --- | --- | --- |
| **Raw data** |  |  |  |
| Total bp Count | 442,721,311 | 520,614,939 | 297,972,766 |
| Sequences Count | 2,033,111 | 2,197,922 | 1,330,983 |
| Mean Sequence Length (bp) | 217 ± 96 | 236 ± 116 | 224 ± 97 |
| Mean GC percent | 52 ± 14 % | 51 ± 15 % | 61 ± 10 |
| Artificial Duplicate Reads: Sequence Count | 21,145 | 23,683 | 29,314 |
| **MG-RAST analysis** |  |  |  |
| Total bp Count | 206,142,758 | 220,119,773 | 136,274,641 |
| Sequences Count | 1,392,411 | 1,319,159 | 899,664 |
| Mean Sequence Length (bp) | 148 ± 73 | 166 ± 90 | 151 ± 74 |
| Mean GC percent | 53 ± 14 % | 51 ± 15 % | 61 ± 10 |
| **Processed** |  |  |  |
| Predicted Protein Features | 1,043,766 | 653,064 | 425,32 |
| Predicted rRNA Features | 25,209 | 25,349 | 13,982 |
| **Annotation** |  |  |  |
| Identified Functional Categories | 253,625 | 129,529 | 150,384 |
