# Supplementary material for: Planktonic microbial profiling in water samples from a Brazilian Amazonian reservoir
Source: Microbiologyopen. 2018 Jan 30;7(2):e00523. doi: 10.1002/mbo3.523 (PMC5911997; doi:10.1002/mbo3.523)

**Supporting information 2**

**Contigs statistics**

|  | Shotgun  Dry period  sample |   Shotgun 1  Rainy period  sample |
| --- | --- | --- |
| N75 (bp) | 319 | 374 |
| N50 (bp) | 419 | 524 |
| N25 (bp) | 583 | 878 |
| Minimum (bp) | 100 | 100 |
| Maximum (bp) | 9,772 | 56,494 |
| Average (bp) | 402 | 476 |
| Count | 108,307 | 129,305 |
| Total (bp) | 43,565,158 | 61,577,195 |

**Gene trees from partial coding sequences of contigs from Dry sample. A) (McyC), B) NRP synthetase, and C) Peptidase S8 .**

A) NJ tree: 427 amino acids alignment based on eight sequences of McyC protein from Microcystis and translated contig 2776. GeneBank accessions are indicated in the nodes. Bootstrap values are indicated in gray.


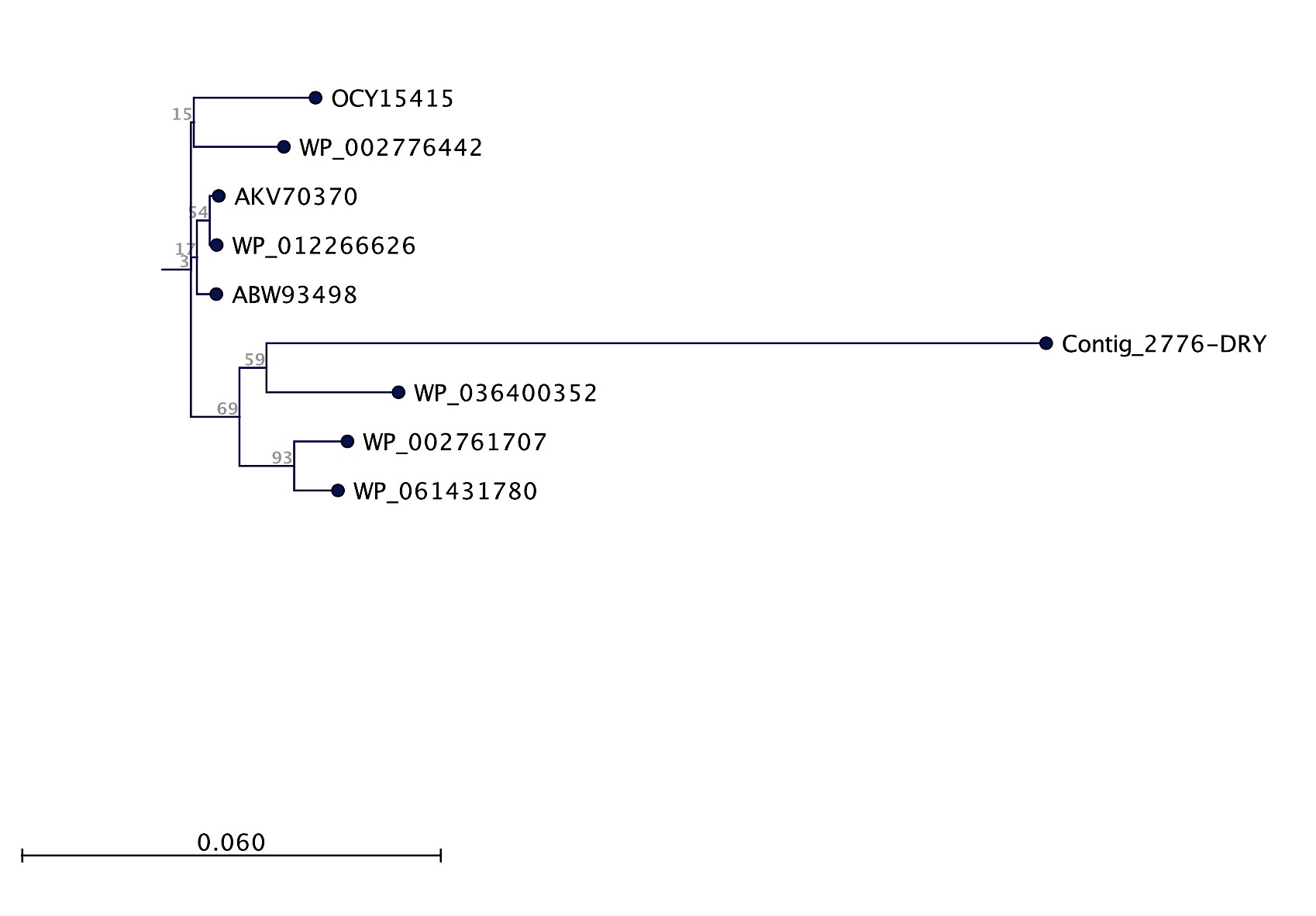


B) NJ tree: 289 amino acids alignment based on nine sequences of non-ribosomal peptide synthetase from Microcystis and translated contig 7916. GeneBank accessions are indicated in the nodes. Bootstrap values are indicated in gray.
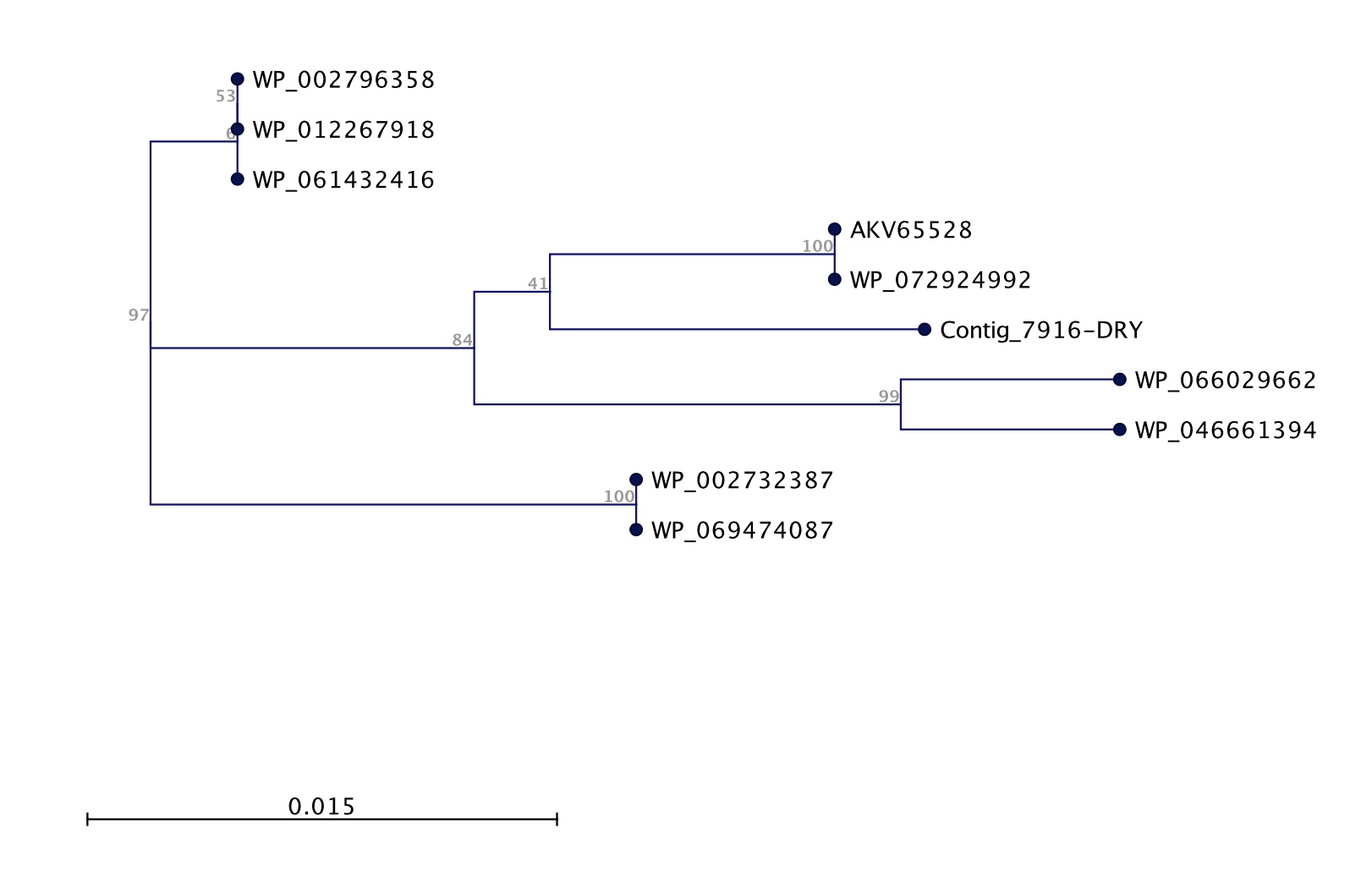


C) NJ tree: 698 amino acids alignment based on nine sequences of peptidase S8 from Microcystis and translated contig 6411. GeneBank accessions are indicated in the nodes. Bootstrap values are indicated in gray.


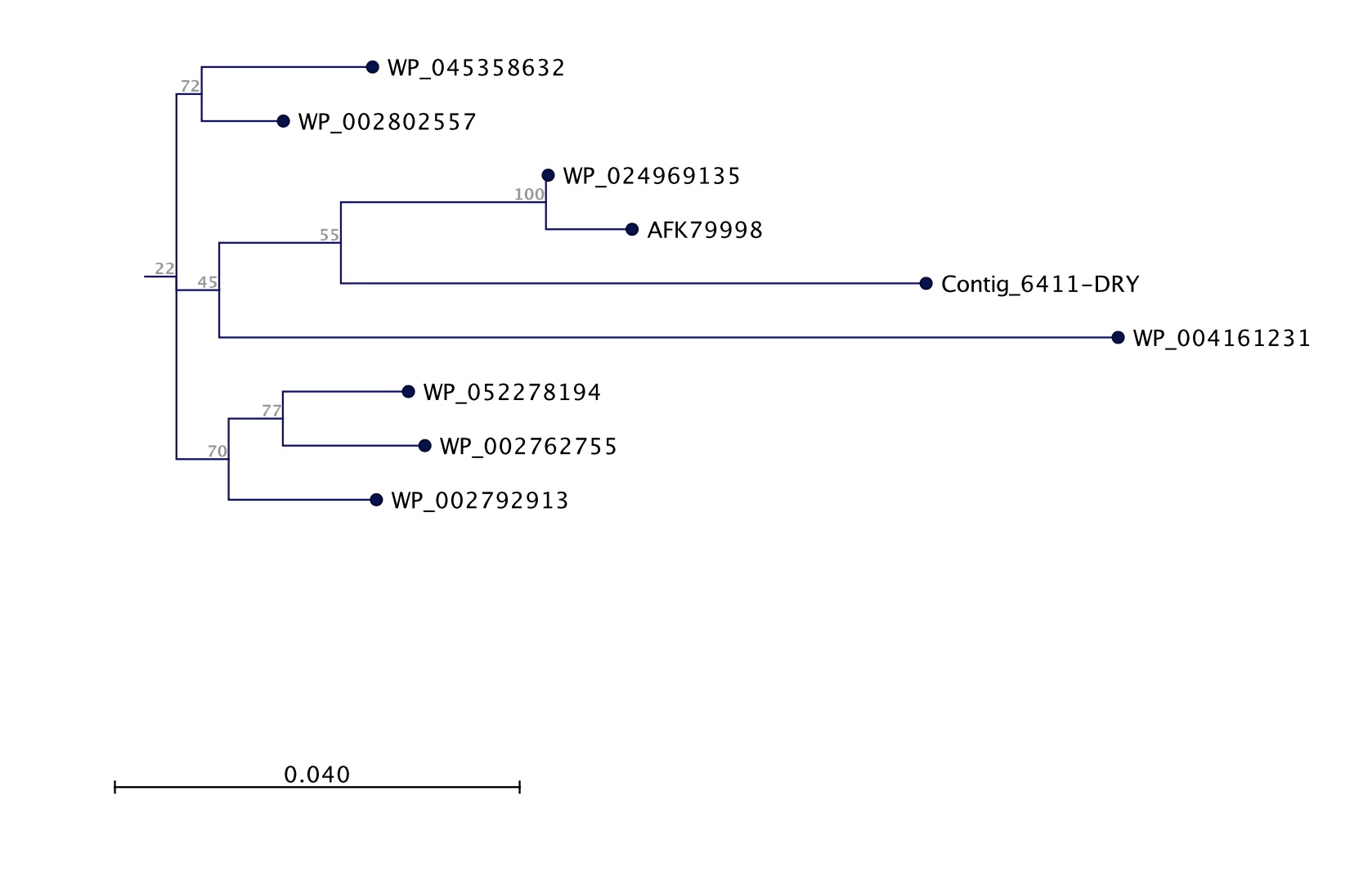

Supplement: Supplementary file 2 [file MBO3-7-na-s002.docx]
